# Therapeutic Potential of Wharton’s Jelly Mesenchymal Stem Cells for Diabetes: Achievements and Challenges

**DOI:** 10.3389/fcell.2020.00016

**Published:** 2020-01-29

**Authors:** Mohamed M. Kamal, Dina H. Kassem

**Affiliations:** ^1^Pharmacology and Biochemistry Department, Faculty of Pharmacy, The British University in Egypt, Cairo, Egypt; ^2^The Center for Drug Research and Development, Faculty of Pharmacy, The British University in Egypt, Cairo, Egypt; ^3^Biochemistry Department, Faculty of Pharmacy, Ain Shams University, Cairo, Egypt

**Keywords:** mesenchymal stem cells, diabetes mellitus, umbilical cord, Wharton’s jelly, insulin producing cells, pancreatic β-cells, regenerative medicine

## Abstract

Diabetes mellitus (DM) is an alarming metabolic disease in which insulin secreting β-cells are damaged to various extent. Unfortunately, although currently available treatments help to manage the disease, however, patients usually develop complications, as well as decreased life quality and increased mortality. Thus, efficient therapeutic interventions to treat diabetes are urgently warranted. During the past years, mesenchymal stem cells (MSCs) have made their mark as a potential weapon in various regenerative medicine applications. The main fascination about MSCs lies in their potential to exert reparative effects on an amazingly wide spectrum of tissue injury. This is further reinforced by their ease of isolation and large *ex vivo* expansion capacity, as well as demonstrated multipotency and immunomodulatory activities. Among all the sources of MSCs, those isolated from umbilical cord-Wharton’s jelly (WJ-MSCs), have been proved to provide a great source of MSCs. WJ-MSCs do not impose any ethical concerns as those which exist regarding ESCs, and represent a readily available non-invasive source, and hence suggested to become the new gold standard for MSC-based therapies. In the current review, we shall overview achievements, as well as challenges/hurdles which are standing in the way to utilize WJ-MSCs as a novel efficient therapeutic modality for DM.

## Introduction

Diabetes mellitus (DM) is a devastating metabolic disease in which insulin secreting β-cells in the islets of Langerhans are damaged to different extent. DM occurs when the pancreas fails to produce and secrete sufficient insulin for the maintenance of glucose homeostasis. Unfortunately, the number of patients suffering from DM is rapidly growing which makes it the most prevalent metabolic disease. Nowadays, about 463 million people worldwide are suffering DM with expected increase to about 700 million by the year 2045 (IDF, 2019).

In fact, cell therapy treatment options for diabetic patients are under extensive study. However, limitations of islet transplantation as a cell replacement approach include the unavailability of the donors, the immune rejections and use of immunosuppressive drugs which have several adverse effects and some of them could damage β-cells (Bhonde et al., 2014). Therefore, these factors strongly encourage the consideration of novel therapeutic interventions such as stem cells’ therapies.

Actually, the field of regenerative medicine is rapidly evolving, paving the way for novel therapeutic interventions through cellular therapies which are indeed reshaping the biomedical field (Pileggi, 2012). During the past years, mesenchymal stem cells (MSCs) specifically have made their mark as a potential weapon in various regenerative medicine applications. In fact, during the past decade, there has been a major leap in understanding MSCs due to unraveling many of their exceptional characteristics, as well as the encouraging data from preclinical and clinical studies (Han et al., 2019). Among the various sources of MSCs, umbilical cord (UC) has proved to be a unique source of MSCs, providing several advantages over other sources (El Omar et al., 2014). It is noteworthy that MSCs have been reported to be present in both UC blood (UCB), and UC tissue/matrix – Wharton’s jelly (WJ). Nevertheless, they are much more abundant in Wharton’s Jelly tissue (Shetty et al., 2010; Arutyunyan et al., 2016). When comparing the success rate for harvesting MSCs, it has been reported to be about 6% from UCB compared to almost 100% from WJ tissue (Shetty et al., 2010). The current review will highlight the achievements, as well as the challenges facing the application of WJ-MSCs as a novel therapeutic modality for DM. Such deep understanding is indeed essential to help the scientific community to overcome those challenges and maximize the therapeutic benefit of WJ-MSCs not only for DM, but also for other disorders.

## Umbilical Cord Wharton’s Jelly as a Potential Source for Stem Cells

During pregnancy, the placenta and growing fetus are connected by UC. This cord prevents umbilical vessels from kinking, compression, or torsion during movement of the fetus, thus ensuring proper blood supply to the fetus (Kim et al., 2013). Anatomically, the human UC comprises an outer layer of amniotic epithelium enclosing a vein and two arteries embedded within a mucoid connective tissue. The mucoid connective tissue enclosing the three umbilical vessels or the UC matrix is known as “Wharton’s jelly.” It was first described by Wharton (1656), and is primarily made of collagen and proteoglycans. The discovery that WJ provides a source of MSCs was first highlighted in McElreavey et al. (1991). Not until 2004 when the first report providing robust evidence that WJ-stromal cells can be classified as MSCs was published (Wang et al., 2004).

First discovered in the early seventies, MSCs, a population of plastic-adherent, non-hematopoietic, fibroblast-like cells, were first isolated from bone marrow (BM) (Friedenstein et al., 1970, 1974). Afterward, in 1991, the term “mesenchymal stem cell” also known as *“multipotent mesenchymal stromal cells,”* was proposed based on their properties (Caplan, 1991; Horwitz et al., 2005). In 2006, the International Society for Cellular Therapy (ISCT) defined plastic adherence, expression of mesenchymal markers while lacking hematopoietic markers and ability to differentiate into osteogenic, adipogenic, and chondrogenic lineages as minimal criteria for definition of MSCs (Dominici et al., 2006). So far, MSCs have been isolated from various tissues including adult tissues such as BM, adipose tissue, liver, as well as fetal/perinatal sources like UCB, placenta, and UC matrix (Da Silva Meirelles et al., 2006; Ma et al., 2014). MSCs were proved to have a broad differentiation potential and several lines of evidence support the notion that these cells may cross germinative layers’ borders being able to differentiate toward ectoderm-, mesoderm-, and endoderm- derived cell types (Nagai et al., 2007; Anzalone et al., 2011).

Interestingly, WJ-MSCs have exceptional properties in that although they are bona fide MSCs (Troyer and Weiss, 2008), possessing similar properties like their adult BM counterparts, yet, they also retain characteristics of primitive stem cells, like the expression of ESC markers (Fong et al., 2011). They may be representing some intermediate state between adult and embryonic stem cells.

In fact, WJ-MSCs have several advantages over adult MSCs in general. They are easily isolated from UC which is readily available; the UC is considered a medical waste discarded at birth. Thus, unlike BM-MSCs which require painful BM-aspiration, the isolation of WJ-MSCs is non-invasive. Moreover, several reports showed a relatively high expression of pluripotency markers in WJ-MSCs compared to MSCs from other sources, implying a more primitive status (Fong et al., 2011; El Omar et al., 2014). Actually, the transcriptomic profile of WJ-MSCs in comparison to other MSCs is reviewed in detail in a comprehensive review article by El Omar et al. (2014). Most recently, an interesting report showed that WJ-MSCs exhibit a unique gene expression profile compared to BM-MSCs using the high throughput single-cell RNA-sequencing technique. In that report, 436 genes were found to be significantly differentially expressed when comparing the two cell types. Those genes are related to several processes such as chemotaxis, apoptosis, anti-tumor activity, and immuno-modulation. The authors reported that those differences might at least in part explain many of the advantages which WJ-MSCs have over BM-MSCs (Barrett et al., 2018).

Furthermore, WJ-MSCs being isolated from neonatal tissue, they may have retained some primitive characteristics similar to ESC. However, unlike ESCs, WJ-MSCs have no ethical concerns (Hass et al., 2011). Moreover, luckily they do not form teratomas upon transplantation (Rachakatla et al., 2007; Troyer and Weiss, 2008; Gauthaman et al., 2012). This can be explained by their unique transcriptomic profile compared to ESCs. WJ-MSCs have been reported to express low levels of pluripotency markers like POU5F-1, SOX-2 and NANOG as compared to ESCs which explains why they do not develop teratomas (Fong et al., 2011).

Moreover, WJ-MSCs have been particularly found to be immune-privileged after reporting their expression of human leukocyte antigen-G (HLA-G) besides their lack of expression of human leukocyte – antigen D-related (HLA-DR) like other types of MSCs (La Rocca et al., 2009). This suggests an immunosuppressive role for these cells mimicking the process occurring *in vivo* at the fetus-maternal interface (Moffett and Loke, 2003). Additionally, WJ-MSCs have a great potential for banking like their counterparts isolated from UCB whose banking nowadays is a very common practice (Chatzistamatiou et al., 2014). Taking in consideration all the interesting findings concerned with WJ-MSCs, it has become indeed tempting to nominate them to become the new gold standard for MSCs-based therapies (El Omar et al., 2014).

## Therapeutic Properties and Mechanisms of WJ-MSCs in Diabetes

Over the past couple of decades, MSCs have indeed made their mark as promising candidates for a wide array of regenerative medicine applications. Originally, MSCs were thought to mediate tissue and organ repair by the virtue of a multilineage differentiation potential that enabled them to replace damaged cells (Mahmood et al., 2003; Murphy et al., 2003). Subsequent findings suggest that in response to tissue injury, MSCs home to the site of damage and encourage repair through the production of trophic factors, including growth factors, cytokines, and antioxidants (Chen et al., 2008; Karp and Leng Teo, 2009), some of which provide the basis for their capacity to modulate immune responses (English et al., 2010). In fact, before carrying out tissue repair functions, MSCs are believed to first prepare the microenvironment by modulating inflammatory processes and releasing various growth factors in response to the inflammation status (Ma et al., 2014).

**FIGURE 1:**
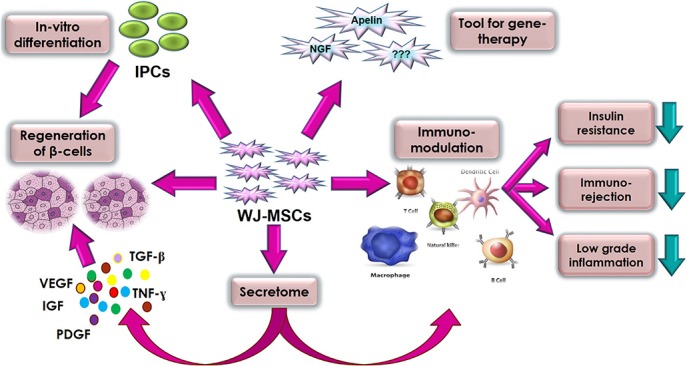
Summary of the various therapeutic properties and mechanisms of action of WJ-MSC in diabetes mellitus.

Generally, clinical applications of MSCs can be attributed to five important biological properties: (a) homing to sites of inflammation following tissue injury when injected intravenously; (b) the secretion of multiple bioactive molecules capable of stimulating recovery of injured cells and inhibiting inflammation; (c) modulating the immune functions (Anzalone et al., 2018), (d) differentiation into various cell types (Wang et al., 2012), and finally (e) as a tool for gene therapy. WJ-MSCs almost use all these properties as a therapeutic potential against DM. We tried to highlight these prominent therapeutic properties and mechanisms of WJ-MSCs related to DM in Figure 1.

### Homing of WJ-MSCs

One of the major characteristics of MSCs is that they have migratory abilities (Nagyova et al., 2014) that is, after administration, they specifically migrate to the sites of inflammation and tissue damage which is typically associated with cytokine out-burst (Sohni and Verfaillie, 2013; Zachar et al., 2016). This can be referred to as “homing”. Actually, the definition of MSCs “homing” is still vague and non-mechanistic. According to a review by Nitzsche and colleagues, they would recommend the definition of homing as the active or passive arrest of a MSC within the vasculature followed by transmigration across the endothelium (Karp and Leng Teo, 2009; Nitzsche et al., 2017).

Stem cells’ homing turned out to be more complicated than once expected and the exact mechanism is far from complete elucidation. It is generally assumed to follow the same pattern as leukocyte homing which includes cell contact with the endothelium by tethering and rolling. Usually the adhesion molecules and selectins are involved in this step (Sackstein, 2004). Thorough understanding of homing mechanisms and factors affecting this homing either intrinsic inside the cells or extrinsic inside the host is crucial for the efficacy and safety of the MSCs administration. Homing is involved in either the systemic administration of the MSCs through intravenous injection, known as systemic homing. This is a multistep process composed of three distinctive phases: (a) direct administration into the circulation, (b) extravasation at the lesioned tissue vicinity, and (c) interstitial migration toward the target site (Deak et al., 2010). Also, local administration of MSCs involves non-systemic homing which requires either recruitment of local MSC or transplantation of exogenous cells close to the target area within the tissue (Nitzsche et al., 2017). This is especially important as it is now established that factors such as age or passage of the cells (Rombouts and Ploemacher, 2003), source and culturing conditions of MSCs (Wynn et al., 2004; De Becker et al., 2007) and even the host pathological conditions and inflammatory milieu (Schenk et al., 2007), affect the homing capacity and therapeutic efficiency of transplanted cells.

Many studies showed that systemic administration of MSCs from different sources showed an initial entrapment of these cells in different organs such as lungs, spleen, liver and even long bones with subsequent distributions from these organs to different tissues (Gao et al., 2001; Bentzon et al., 2005). Gao et al. showed that following either intra-arterial or intra-venous radioactive labeled MSCs infusion, MSCs were detected primarily in the lungs and then secondarily in the liver and other organs, an effect that was enhanced with vasodilators Gao et al. (2001). This can be associated with huge reduction in the number of cells reaching the target site of injury. Also, this might be associated with unknown effects of these cells on such organs. Actually, it was shown that entrapment of MSCs in liver and spleen may be associated with suppression of T-cell activation (Jellema et al., 2013). Usually the cells could be injected either intravenously (IV) or intra-arterially (IA) for systemic infusion. Usually the IV is the safest, least invasive and easiest to perform, however, it is associated with cells entrapment in the lungs (De Becker and Riet, 2016). On the other hand, the IA injection is more invasive and may be associated with risk of development of microvascular occlusions which is known as passive entrapment (Karp and Leng Teo, 2009). Several excellent reviews have been discussing these MSCs dynamics issues (De Becker and Riet, 2016; Zachar et al., 2016; Nitzsche et al., 2017).

Unfortunately, the study of the homing mechanisms of the WJ-MSCs in general and in diabetes particularly, represents a clear gap of knowledge. One recent study showed that like other MSCs, homing abilities have been reported for injected exogenous WJ-MSCs. An interesting study injected labeled WJ-MSCs into type 2 DM animal model as well as control non-diabetic animals, through the tail vein. In that study, transplanted WJ-MSCs were detected in lung, liver, and spleen in both normal and diabetic mice. However, unlike normal mice, a certain number of WJ-MSCs also homed to the pancreatic islets of diabetic mice, suggesting that homing of WJ-MSCs is closely related to tissue damage (Yin et al., 2018). However, still further studies involving homing of WJ-MSCs are warranted.

### Secretome and Paracrine Actions of WJ-MSCs

Interestingly, a growing body of research has revealed that the therapeutic effects of stem cells occur largely via paracrine signaling, and secreted extracellular vesicles/exosomes (Nooshabadi et al., 2018). In agreement with that belief, several studies reported beneficial therapeutic effects of WJ-MSCs-conditioned media (Arno et al., 2014; Fong et al., 2014). Most recently, WJ-MSCs have been reported to mediate their pro-angiogenic activities via secretion of angiogenin, interleukin-8, monocyte chemoattractant protein-1, and vascular endothelial growth factor. The association between their therapeutic pro-angiogenic potential and the secreted levels of those factors was observed in *in vitro* angiogenesis assays, as well as *in vivo* in a rodent model of hind limb ischemia. Moreover, knocking down the expression of those factors by siRNA significantly inhibited the pro-angiogenic activities of WJ-MSCs. Additionally, they found that donor-to-donor variation was commonly observed in WJ-MSCs regarding their secretome profiles, even when using identical culture conditions and passage number (Kim et al., 2019).

Furthermore, paracrine effects of WJ-MSCs have been reported for neuronal regeneration. WJ-MSC-conditioned medium has been reported to enhance Schwann cell viability and proliferation (Guo et al., 2015). Also, BDNF and hepatocyte growth factor (HGF) secreted by WJ-MSCs have been reported to exert neuroprotective effect on damaged neurons (Mukai et al., 2018). In addition, WJ-MSCs conditioned medium has been reported to contain several secreted factors mediating their immune modulatory effects such as IL-2, IL-6, IL-8, IL-12, IL-15, MCP-1, MIP-1, RANTES as well as prostaglandin E2 (PGE2) (Yoo et al., 2009).

Interestingly, WJ-MSCs were also suggested to change their secretome profile based on the context of a lesion or generally the conditions to which they are exposed (Carvalho et al., 2011). In accordance with that, its noteworthy here that most recently, gestational DM has been reported to significantly affect the stemness properties, differentiation potential, oxidative stress, senescence and mitochondrial function of WJ-MSCs of UCs of mothers with gestational DM (Kong et al., 2019).

In addition, WJ-MSCs may mediate their paracrine actions through their extracellular vesicles among which are exosomes. Exosomes are defined as nano-sized bioactive vesicles derived from the cell’s endosomal membrane system. They act as shuttles transferring specific cargos of proteins, mRNA, as well as non-coding RNAs like microRNA; as a message in a bottle. Accordingly, they can reprogram the recipient cells and are regarded as “signalosomes” controlling fundamental cellular functions (Bjorge et al., 2017).

In fact, the utilization of WJ-MSCs secretome as a cell-free potentially effective regenerative medicine tool especially for allogenic therapeutic applications is indeed tempting. However, that secretome should be well-characterized and elucidated in detail in order to be utilized properly. Provided that we are witnessing the “OMICS” era, further large-scale proteomics and metabolomics studies are indeed warranted to unravel the exact components of WJ-MSCs secretome, and the mechanisms regulating the secretion of various trophic and immuno-regulatory factors by WJ-MSCs.

### Immunomodulatory Effects of WJ-MSCs

Another important feature of WJ-MSCs that actually augments their clinical utility is their low immunogenicity. This is due to the fact that they express MHC class I (HLA-ABC) at low levels but neither class II (HLA-DR) nor co-stimulatory antigens such as CD80, CD86 implicated in activation of both T and B cell responses (Pappa and Anagnou, 2009; Prasanna et al., 2010). In comparison to BM-MSCs, WJ-MSCs does not express HLA-DR upon IFN-γ stimulation. Besides, WJ-MSCs produce huge amounts of immunosuppressant cytokines such as IL-10, TGF-β, IL-6 and VEGF, which have recently been shown to be pivotal in the immunosuppressive capability of MSCs (Weiss et al., 2008). These properties make WJ-MSCs less immunogenic than BM-MSCs making them more amenable for allogeneic transplantation.

Moreover, WJ-MSCs were found to affect almost all cells of the immune system. MSCs, including WJ-MSCs, can suppress the CD3, CD4, and CD8 T-cells (Aggarwal and Pittenger, 2005). Interestingly, WJ-MSCs can suppress allogenic-stimulated immune cells to a greater extent than either BM-MSCs or adipose-derived MSCs (Kim et al., 2013). In addition, WJ-MSCs inhibits the maturation and activation of dendritic cell (DC) precursors by locking the monocytes in an immature DC phenotype (Tipnis et al., 2010). Besides, WJ-MSCs can affect the functions of NK cells (Casado et al., 2013), monocyte/macrophages (Cutler et al., 2010) and even neutrophils and mast cells (Brown et al., 2011). Although many details of these interactions remain to be elucidated, yet, both cell-to-cell contact and soluble factors are thought to be key aspects of WJ-MSCs-mediated immunosuppression (Shi et al., 2010; Ma et al., 2014). These immuno-modulatory effects are the basis of their utility in several diseases such as graft versus host disease (GvHD) (Newell et al., 2014), and DM (Katuchova et al., 2015).

It is noteworthy here to highlight that WJ-MSCs have been reported to exert systemic effects in type 2 DM animal models by modulating macrophages in immune organs, and adipose tissue via increasing M2 (anti-inflammatory) macrophages, causing profound anti-diabetic effects and enhanced insulin sensitivity (Xie et al., 2016; Yin et al., 2018).

### Differentiation and Cell Replacement Potential of WJ-MSCs

In addition to immunomodulatory functions and paracrine signaling, the differentiation potential of WJ-MSCs down several lineages adds a lot to their therapeutic potential, not only for DM, but also for a wide array of other diseases (Can et al., 2017). In fact, several pre-clinical studies reported the differentiation potential of WJ-MSCs into insulin producing cells (IPCs) using various induction protocols, and inducing extrinsic factors (Kassem et al., 2016; Pavathuparambil Abdul Manaph et al., 2019).

Additionally, many studies proved the capacity of both differentiated and undifferentiated WJ-MSCs to regulate hyperglycemia in several DM animal models. Interestingly, in order to find out whether undifferentiated WJ-MSCs can differentiate into pancreatic IPCs or not when injected into animal model of DM. Tsai and coworkers, labeled WJ-MSCs using GFP-lentiviral transduction, then injected those cells intravenously into NOD mice. Afterward, co-localization of human C-peptide and GFP was detected in the pancreas; proving that transplanted WJ-MSCs indeed differentiated into IPCs *in vivo* (Tsai et al., 2015). Furthermore, another report highlighted the effect of intravenous infusion of human WJ-MSCs as a therapy by administering them in a type 2 DM rat model. The rats treated with WJ-MSCs exhibited increased numbers of β-cells; suggesting the therapeutic potential of WJ-MSCs in β-cell regeneration (Hu et al., 2014). Moreover, a recent study compared the anti-diabetic regenerative potential of WJ-MSCs compared to BM-MSCs in diabetic rats. WJ-MSCs showed better anti-diabetic effects and potential for pancreatic regeneration (Som and Venkataramana, 2018). WJ-MSCs were also proved better than UCB-MSCs in the same aspect (El-Demerdash et al., 2015).

However, it’s important to highlight here that growing body of evidence support the notion that MSCs mostly mediate their therapeutic effects via their secretory/paracrine and immunomodulatory functions rather than their *trans-*differentiation potential *in vivo* (Päth et al., 2019).

### WJ-MSCs as a Tool for Gene Therapy

Most recently, an interesting study transduced WJ-MSCs with apelin-expressing lentiviral particles, and those genetically-modified cells were injected into type 2 diabetes rodent model. Infusion of these WJ-MSCs-apelin significantly improved insulin sensitivity, and increased the levels of plasma C-peptide. Moreover, the serum levels of inflammatory cytokines TNF-α and IL-6 decreased, and anti-inflammatory adiponectin levels increased. Also, endogenous pancreatic β-cell proliferation profoundly increased more than 9 folds in the treated group compared to the control group (Gao et al., 2018). Furthermore, WJ-MSCs overexpressing nerve growth factor (NGF) were reported to induce neural regeneration, and to ameliorate symptoms of diabetic cysto-pathy in diabetic rats (WenBo et al., 2017). These reports imply the strong potential of WJ-MSCs to be used as a tool for various gene-therapy applications.

All these therapeutic properties of WJ-MSCs set the stage for these cells to find their ways to clinical trials. In the next section of this review, we will discuss how far these clinical trials went down the way of DM therapy.

## Clinical Application of WJ-MSCs in Diabetes

Over the last years, various clinical trials have been conducted during the past decade to test the feasibility and efficacy of WJ-MSCs therapy for various disease conditions such as: neurological disorders, cancer, cardiac disease, liver disease, bone/cartilage disease, immunological diseases as well as DM and its complications. Many of these clinical trials have been completed and indeed demonstrated the safety and efficacy of WJ-MSCs (Can et al., 2017). Actually, the public clinical trials database is currently showing about 980 trial investigating MSCs from various sources for a wide array of therapeutic applications; 64 of these trials are related to DM and its complications *(Accessed on August, 2019)* (ClinicalTrials.gov, 2019).

Regarding the 64 studies currently registered on the public clinical trials database *(Accessed on August, 2019)* investigating MSCs (from various sources) for treating DM and/or its complications, only about 16 of these studies clearly state applying WJ-MSCs as an intervention (ClinicalTrials.gov, 2019). However, unfortunately, many of them are having “unknown-status” labeling, or no reported results. Thus, we tried to summarize the most prominent published completed clinical studies investigating the therapeutic potential of WJ-MSCs for DM together with their reported outcome in Table 1. It is noteworthy here, that these are different from other clinical studies utilizing MSCs or even hematopoietic stem cells (HSCs) derived from UCB, which is out of the scope of the current review.

**TABLE 1:** Clinical studies applying WJ-MSCs for diabetes mellitus.

**Patient criteria**	**Number of cells**	**Route of delivery**	**Therapeutic outcome**	**Adverse effects**	**References**
Type 1 DM – newly onset 29 patients divided into 2 groups: 15 patients received WJ-MSCs and 14 patients received normal saline (control group); randomized double blind placebo-controlled study	1.5–3.2 × 10^7^	IV – Parenteral solution Twice, 4 weeks interval	Patients were followed up for 24 months HbA1c and C-peptide values significantly improved	–	Hu et al., 2013
Type 2 DM 22 patients received WJ-MSCs transplantation Non-placebo controlled phase I/II study	1 × 10^6^/kg	1 IV and 1 Intra-pancreatic endovascular injection, 5 days interval	Patients were followed up for 12 months HbA1c and glucose levels significantly decreased. Improved C-peptide levels and β-cell function. In addition to reduced systemic inflammation and T lymphocyte counts	Mild fever in 3 of 22 patients on the first operative day Nausea, vomiting and headache in 1 patient, spontaneously recovered within 1 week.	Liu et al., 2014
Type 2 DM 18 patients received WJ-MSCs transplantation	Not specified	3 IV doses	Patients were followed up for 6 months Significant decrease in fasting and post-prandial blood glucose levels, and increase of C-peptide levels and Tregs	4 out of 18 patients had transient slight fever	Kong et al., 2014
Type 1 DM – long standing (2–16 years duration) 21 patients received stem cell therapy (and additional 21 patients acting as control group who did not receive neither stem cell intervention nor placebo).	1.1 × 10^6^/kg WJ-MSC –allogenic plus 106.8 × 10^6^/kg BM-MNC – autologous	Intra-pancreatic	Patients were followed up for 12 months Significant decrease in FBG and HbA1c levels, increased C-peptide and insulin levels. Significant reduction in insulin dose requirement	Transient abdominal pain observed in 1 patient and spontaneously resolved, and 1 patient with puncture site bleeding resolved with pressure	Cai et al., 2016
Type 2 DM 31 patients received WJ-MSCs and 30 patients received normal saline (control group); randomized double blind placebo-controlled study	1 × 10^6^/kg	IV – Parenteral solution Twice, 4 weeks interval	Patients were followed up for 36 months Blood glucose and HbA1c levels significantly decreased. C-peptide levels and pancreatic β-cell function significantly improved. In addition to reduction of insulin and oral hypoglycemic agents dose requirement Reduced incidence of diabetic complications	No serious adverse events reported.	Hu et al., 2016
Diabetic foot 28 patients received WJ-MSCs and 25 patients acting as control group (did not receive WJ-MSCs intervention); randomized study	4.8–8.6 × 10^7^ cells	Endovascular infusion and injection around the foot ulcer – Local transplantation	Patients were followed for 3 months Improvements in skin temperature, ankle-brachial pressure index, transcutaneous oxygen tension, and claudication distance. Significant increase in neo-vessels, accompanied by complete or gradual ulcer healing.	–	Qin et al., 2016
Type 2 DM – more than 10 years duration 12 patients were enrolled and randomly divided into two groups: - 6 patients receiving Liraglutide plus WJ-MSCs intervention - 6 patients receiving Liraglutide only (control group) (Article in Chinese)	1 × 10^6^ cells/kg	Intra-pancreatic artery infusion on the first day followed by IV infusion on the 8^th^, 15^th^, and 22^nd^ day sequentially	Patients were followed for 6 months Significant decrease in FBG, post-prandial glucose and HbA1c levels Significant improvements in C-peptide and HOMA-IR levels	–	Chen et al., 2016

In a clinical study by Hu and coworkers, Out of the 15 treated patients, 3 became insulin independent, and in 8 out of the remaining 12 patients, the daily insulin requirements were reduced by more than 50% compared to their starting baseline (Hu et al., 2013). On the other hand, Liu and coworkers applied WJ-MSCs transplantation in type 2 DM patients with disease duration of about 8.7 ± 4.3 years, and they demonstrated that treatment with WJ-MSCs can indeed improve metabolic control and β-cell function. They also suggested that their mechanism of action may have involved improvements in systemic inflammation as well (Liu et al., 2014). Thus, WJ-MSCs proved to be safe and relatively effective in both type 1 and 2 DM, as well as recently diagnosed or relatively long-standing diabetic patients.

Most recently, an interesting double-blinded randomized placebo-controlled trial has been inaugurated in Sweden and started recruiting patients since January 2018 with estimated measurement of primary outcome in 2020. That study aims to investigate the safety and efficacy of allogenic WJ-MSCs as an investigational medicinal product produced under GMP conditions with minimal batch-to-batch variation (Carlsson and Svahn, 2018). Actually, well-designed large scale randomized clinical trials are indeed warranted to consider not only the safety, but also the optimum dosage regimen and treatment protocol to be followed.

In fact, several meta-analysis studies have been carried out over the past few years attempting to figure out the efficacy of stem cell therapy in DM, and where we are standing exactly. Among these, an interesting comprehensive meta-analysis by El-Badawy and El-Badri included 22 studies investigating stem cell therapy for DM, among which six studies used MSCs. Interestingly, they reported that infusion of WJ-MSCs provided significantly better therapeutic outcome in type-1 DM when compared to BM-MSCs. While BM-mononuclear cells (MNCs) infusion provided better outcome compared to WJ-MSCs in type-2 DM patients. Additionally, they also found that administration of stem cell therapy for recently diagnosed DM was more effective than intervening at later stages. Yet, it’s important to keep in mind that those findings are not conclusive and were based on relatively small number of trials and requires confirmation via larger trials (El-Badawy and El-Badri, 2016).

It is important to highlight here that actually most if not all of the clinical studies employing WJ-MSCs are still in early phase I or II. Thus, despite the young age of WJ-MSCs and their ever-growing list of advantages over those isolated from older adult tissue sources like BM or adipose tissue (Kim et al., 2013; El Omar et al., 2014), yet, looks like there is still a long way to go for those cells to reach actual clinical application, and to cut the way from bench to bed-side!

In addition, it is noteworthy here that given the immune-privilege of the WJ-MSCs, their good culture conditions and supported by the above-mentioned studies, WJ-MSCs can be considered as a potential candidate for allogenic transplantation. This may be considered a step forward in development of what is called “off-shelf stem cells drugs.” A very recent review addressed this issue and the authors declared that MSCs can effectively be used as an allogenic off-shelf drug despite special concerns of their HLA make up (Kot et al., 2019). This notion requires further investigations and considerations of the challenges that will be further discussed in this review.

## Challenges for WJ-MSCs Translation From Bench to Bed-Side

### Safety Issues

When considering WJ-MSCs as a novel therapeutic intervention not only for DM, but also for other disease conditions, the majority of clinical trials reported the safety and absence of serious acute or chronic adverse effects. Occasionally, transient fever, headache or local pain were reported, yet those adverse events were also reported to resolve spontaneously within few days after transplantation (Can et al., 2017). However, when thinking of WJ-MSCs, being after all a type of stem cells expressing pluripotency factors, and also being applied for allogenic transplantations; in other words, foreign cells supposed to reside and live in the hosting patient/human body for a long term. At that moment, several safety concerns pop up. Such as the possibility of immuno-rejection by the recipient host, the genetic stability, as well as the possible tumorigenicity of those cells.

Actually, what makes MSCs, and especially WJ-MSCs an ideal candidate for regenerative medicine, with potential capabilities not only to regenerate damaged tissues but also to reduce rejection possibilities are their unique immunomodulatory properties (English et al., 2010; Can et al., 2017). These properties provide some sort of relief regarding the concern of possible immuno-rejection.

Nevertheless, WJ-MSCs have been reported to exhibit changes in their gene expression and transcriptomic profile, promoted by prolonged *in vitro* culture till the 12^th^ passage compared to the early 4^th^ passage. Those genes with altered expression have been reported to be related to several processes like: proliferation, differentiation, apoptosis, and inflammation. Briefly, WJ-MSCs according to these findings, showed a progressive decline in their physiological properties with prolonged *in vitro* culture; a phenomenon known as *“Cellular aging”* (Gatta et al., 2013). These observations shed light on the crucial importance to keep such possible changes in mind and emphasize the need to carry out essential testing and investigations on the cells to ensure that they are functioning properly and not tumorigenic before their transplantation to the patient.

As for tumorigenicity concern, it is indeed a key hazard which may arise due to transformed cells present in the final administered product, or even possibly developed as a long-term adverse effect of those transplanted cells. Several studies have observed that tumorigenicity may represent a potential adverse effect of MSCs therapy through different mechanisms. First, several studies showed that MSCs may represent the direct cellular origin of cancer, like one study showed that MSCs are source of gastric sarcoma (Kauer et al., 2009; Barkholt et al., 2013; Neri, 2019). Second, MSCs may secrete or produce several inflammatory mediators and chemokines as paracrine factors such as CXCLs or interleukins which were found to support several cancers such as breast or colon cancers (Liu et al., 2011; Tsai et al., 2011). The third mechanism by which MSCs can support tumor formation is the immune suppressive actions of these cells in both innate and adaptive immunity in a way that can promote tumor growth and progression (Lee and Hong, 2017). In fact, several contradictory results have been reported regarding pro- or anti-tumorigenic effects of MSCs. However, as far as our knowledge, so far no cancer was detected in clinical trials using MSCs, a notion supported by relatively recent reviews (Lee and Hong, 2017).

Interestingly, the application of exogenous engineered MSCs has been investigated as a novel anti-cancer strategy (Nowakowski et al., 2016). As for WJ-MSCs specifically, they have been reported to secrete factors causing suppression of cancer growth and inducing apoptosis (Wu et al., 2013; Lin et al., 2014). Generally, WJ-MSCs are reported to be non-tumorigenic, or even anti-tumorigenic and suggested to be a safe promising tool for cancer therapy. But the exact mechanisms of such anti-tumorigenic effect are still far from complete elucidation (El Omar et al., 2014).

### Good Manufacturing Practice (GMP) – Compliance

It is important to remember that WJ-MSCs, like all other cell-based therapies are living products constantly interacting with their surrounding environment. This poses new challenges when looking from a pharmaceutical industrial point of view. For example, they cannot be sterilized prior to use to provide strict protection from any contamination transmission to patients. Besides, in order to maintain a consistent product efficacy, with minimal batch-to-batch variation, this requires precise process control and avoidance of adverse changes in heterogeneous populations or the cell environments. Consistent GMP-compliant manufacturing requires high reproducibility with a focus on safety, efficacy, and quality (Stacey et al., 2018).

Basically, WJ-MSCs are considered an advanced therapy medicinal product (ATMP), and should be produced in compliance with GMP (EMA, 2007). These requirements include the following: (a) Tests for virology (HIV-1/2, HBV, HCV, HTLV-1/2, HPV, B-19, CMV, and EBV), syphilis, mycoplasma, and sterility being negative. (b) Phenotype: the percentages of CD73+, CD90+, and CD105+ cells ≥ 98% and the percentages of CD34−, CD45−, HLA-DR−, CD14− or CD11b−, CD79a−, or CD19−≤2%. (c) Viability ≥ 80% after thawing. (d) The endotoxin content < 2 EU/mL. (e) No significant upregulation of telomerase reverse transcriptase (hTERT) gene and oncogenes during large-scale expansion. (f) No significant downregulation of tumor suppressor genes during large-scale expansion. (g) Confirmed potency (Sensebé et al., 2013; Arutyunyan et al., 2016).

Actually, one of the main concerns regarding using WJ-MSCs for clinical applications is the requirement of *in vitro* expansion which is affected greatly by culture medium. In the same time, production of clinical grade WJ-MSCs requires sterility controls, analysis for viral markers, and genetic testing such as karyotyping. It is important to point here that serum is a very important component of the culture as well as cryopreservation media of WJ-MSCs. It provides nutrients required for the cells’ expansion and survival. However, residual proteins from animal serum might cause serious immunological reactions, which could adversely affect the therapeutic potential of the injected cells (Li et al., 2015).

Several studies have suggested alternatives to medium containing FBS or xenogenic serum (Bieback et al., 2009). Some investigators suggested using human serum or platelet-rich plasma (PRP) as an alternative for FBS (Jonsdottir-Buch et al., 2013). Furthermore, an interesting report suggested using PRP isolated from the same UC source, and indeed reported impressive results. Briefly, they collected UCB and UC-tissues from the same donors. The UC tissue was processed to obtain WJ-MSCs, and UCB was used as a source of activated PRP for WJ-MSCs expansion; as a GMP-compliant protocol for clinical applications (Van Pham et al., 2016).

Also, the cryopreservation method, and the optimum agents that should be used are still under debate (Balci and Can, 2013). Dimethyl sulfoxide (DMSO) has always been used as the common-practice cryo-protective agent; protecting the cells from freezing-induced damage. However, DMSO is potentially toxic especially at temperatures greater than 4°C, which complicates direct use of thawed MSCs for clinical applications. Moreover, the use of frozen-thawed MSCs treated with DMSO has been reported to adversely affect patients and cause possible nausea, vomiting, or even death (Thirumala et al., 2013). Importantly, the composition of the cryo-protectant media and the freezing protocol have been found to greatly affect the efficacy of obtaining living healthy cells from thawed UC-tissues (Arutyunyan et al., 2018).

Finally, maintaining cost effectiveness is indeed a very important crucial aspect to consider for manufacturing companies. And when considering scaling-up for generation of efficient cellular therapies from WJ-MSCs, GMP-compliance together with efficient cost-effectiveness is absolutely one of the major challenges facing the translation of WJ-MSCs from bench to bed-side.

### Dosage Regimen

Effectiveness of stem cell therapy in DM is multifactorial. Efficiency and therapeutic outcome of injected cells are greatly affected by the initial source of those cells, the number of injected cells and the route of administration (El-Badawy and El-Badri, 2016). Accordingly, it is indeed essential to optimize the cell dose and frequency, as well as the best route of delivery for every condition. MSCs over the past 2 decades have been mostly administered intravenously (IV). However, in case of local pathological conditions such as spinal cord injuries, *in situ* administration of cells is generally preferable (Can et al., 2017).

For the 7 clinical studies reviewed in Table 1 which reported beneficial therapeutic effects for WJ-MSCs in DM or DM-related complications, 3 of those studies administered WJ-MSCs solely via IV injection, 1 study transplanted the cells via intra-pancreatic artery (IPA), 2 studies applied both IV and IPA administrations, and the last study injected the cells around the foot ulcer – local transplantation. Interestingly, apart from the route of administration, when considering the dosage regimen, or the frequency of administration, several regimens are also found in literature. Some studies used single dose injection, especially for intra-pancreatic route (Cai et al., 2016). While others used multiple injections ranging from two or more times, separated by varying periods of time (Kong et al., 2014; Chen et al., 2016; Hu et al., 2016). As for the dose/count of the cells to be transplanted, it varied from 0.2 × 10^6^/kg and 8.7 × 10^6^/kg in various disease conditions. Mostly the cell count to be administered is calculated relative to body weight, however, some clinical studies also applied some sort of arbitrary count (not calculated relative to body weight) (Can et al., 2017). Regarding WJ-MSCs transplantation for DM, a dose of 1 × 10^6^/kg was reported several times in the studies reviewed in Table 1.

Actually, it is important to highlight here that the number/count of cells, the best route of delivery, the number/frequency of doses, as well as the time intervals between multiple deliveries are indeed very important controversial issues. Further, large scale well-designed randomized placebo-controlled studies are undoubtedly required to resolve these controversies and reach the optimum dosage regimen that should be followed for different types of DM, and DM-related complications.

**FIGURE 2:**
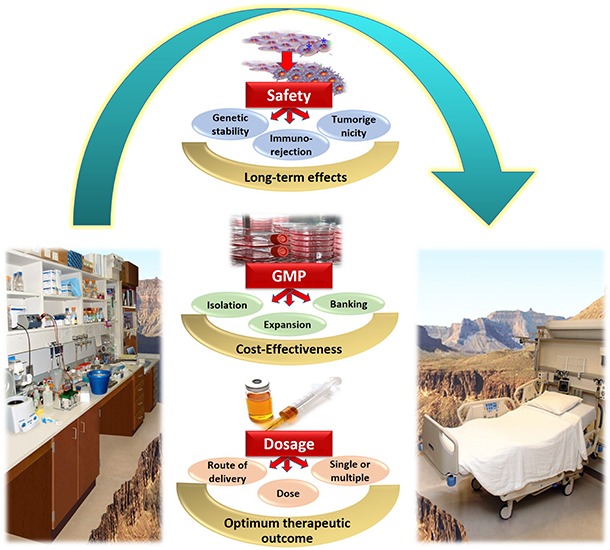
Summary of the challenges and hurdles in the way of utilizing WJ-MSCs for treating diabetes mellitus, and transferring them from bench to bed-side.

In conclusion, several issues concerned with obtaining clinical grade WJ-MSCs, and the transition of WJ-MSCs therapy from bench-side to bed-side are still unresolved. Several challenges need to be well-addressed in order to maximize the benefit of WJ-MSCs for various regenerative medicine applications. Figure 2 provides a summary for the most prominent challenges and hurdles facing WJ-MSCs in order to be translated from bench to bed-side.

## WJ-MSCs Banking and Clinically Related Products

### WJ-MSCs Banks

The estimation of more than 130 million global annual births worldwide indeed provide a unique opportunity as well as a readily available endless supply of life-saving stem cells, which could be recovered from WJ and UCB, then banked for future autologous or allogenic therapeutic application. It has been more than 20 years since the first human UCB transplant was performed (Munoz et al., 2014). Nowadays, UCB-banks worldwide hold 100s of 1000s of stored cryopreserved UCB units. The methods of collection, cryopreservation and banking of UCB are currently well-established, and thus clinical outcomes continue to improve worldwide for various conditions (Shearer et al., 2017).

However, regarding WJ-MSCs, the best practices for their cryopreservation/thawing and banking processes are still not clear, and not well-standardized. On one hand, the Hellenic UCB-bank reported that viable MSCs can only be isolated from freshly isolated UC-WJ-tissue, and that isolation of WJ-MSCs from frozen tissue fragments was impossible and completely unsuccessful (Chatzistamatiou et al., 2014). On the other hand, another research group from Singapore compared the post-thaw behavior of isolated WJ-MSCs, WJ-tissue, and whole/entire UC segments. Interestingly, they found that viability, proliferation rates, and even apoptotic signals of post-thawed cells which were originally cryopreserved as WJ-MSCs, or isolated from freezed-thawed WJ-tissue were significantly better than those cells derived from freezed-thawed whole UC segments. In conclusion, they reported that freezing WJ-tissue is a simple and reliable method for obtaining large numbers of MSCs to be utilized for cell-based therapies (Fong et al., 2016). Its noteworthy that storage of unprocessed WJ-tissues compared to storing isolated WJ-MSCs could have several advantages; such as minimization of labor and time expenses, as well as better chances for cell isolation and expansion in the future with yet unknown future standards or guidelines (Arutyunyan et al., 2018).

Nowadays, several UCB banks worldwide offer the service of UC-tissue cryo-preservation such as *Cryo-Cell, ViaCord, Cells4Life, CellCare, Cryo-Save*, and others (Arutyunyan et al., 2018). In fact, they encourage storing both UC-tissue and UCB, as the most efficient way to store the child’s stem cells. We totally agree with this, taking in consideration the growing body of evidence supporting the regenerative potential of those WJ-MSCs for various disease conditions. However, still the currently used protocols for processing and cryo-preservation of the UC-tissue require careful revision. Standardization of such protocols is indeed warranted to ensure maximum future benefit of these cryopreserved UC-tissues, and to ensure getting clinical grade potentially effective WJ-MSCs for therapeutic applications in the future.

### WJ-MSCs and Other UC-Related Products/Trademarks on the Market

In fact according to the European Medicines Agency (EMA), MSCs are considered as ATMP when these cells undergo substantial manipulation or are used for a different essential function. Actually, due to the diversity and complexity of cell-based biopharmaceuticals, the concept behind ATMP development in the EU – the regulation (EC) No. 1394/2007 lays down specific guidelines concerning centralized authorization, supervision and pharmacovigilance, always keeping in mind a strict focus on risk-assessment toward safety of the end user (EMA, 2007). Among the first attempts to produce an ATMP from WJ-MSCs has been the development of UCX^®^ manufactured by ECBio (Amadora, Portugal). It has been characterized in terms of cell identity, purity (microbiological, identity, and viability), tumorigenicity and genetic stability (Martins et al., 2014). It is among the first attempts to produce an ATMP from WJ-MSCs in accordance with EMA regulations regarding GMP for producing an ATMP in 2013–2014. However, we believe that its applications are still investigational, like WJ-MSCs in general. This product is an example of a potential product derived from UC tissue – WJ-MSCs, produced on a relatively large scale by ECBio company in accordance with GMP requirement for an ATMP.

Another UC-related trademark in the market is EpiCord^®^. EpiCord^®^ is a minimally manipulated, dehydrated, devitalized cellular UC allograft commercially available. It is created through a patented PURION Plus process resulting in an allograft material that can be stored in ambient conditions for 5 years. EpiCord^®^ showed indeed very promising results for treating non-healing diabetic foot ulcers (DFU) (Tettelbach et al., 2019). NEOX^®^ Wound Allograft (Amniox Medical, Atlanta, GA, United States) is another UC-related product in the market. NEOX^®^ is cryopreserved human amniotic membrane and UC (AM/UC) tissue dressing indicated for skin ulcers. Efficacy of NEOX^®^ for treating chronic DFUs has been reported, and impressively 87.5% of wounds achieved complete epithelialization within about 14 weeks (Raphael, 2016).

## Future Perspectives

It’s noteworthy here, that over the last few years, much attention has been given to decide who would be the winner for diabetes remodeling; ESCs or iPSCs? Additionally, several high throughput elegant studies have been focusing on generation of IPCs from ESCs (Pagliuca et al., 2014; Vegas et al., 2016; Jacobson and Tzanakakis, 2018; Nair et al., 2019). But still on the other hand, several elegant studies and review articles attempt to investigate the differentiation potential of MSCs into various lineages other than mesoderm (Urrutia et al., 2019). Insulin producing cells is definitely one of these lineages (Enderami et al., 2018; Päth et al., 2019; Pavathuparambil Abdul Manaph et al., 2019). Accordingly, we recommend that interested scientists should also consider developing novel strategies and enhancing induction protocols to generate IPCs from these WJ-MSCs.

Actually, huge line of evidence showed that WJ-MSCs do not impose any ethical concerns or teratoma risk as those which exist regarding ESCs or IPCs and represent a readily available non-invasive source. Besides, several mechanisms have been suggested and discussed in this review for their therapeutic potential in diabetes including differentiation into IPCs, secretion of paracrine and soluble factors and modulating the immune response in the patient. Hence, they are potential candidates to become the next frontier for DM cell therapy.

Whether WJ-MSCs remain immuno-privileged and maintain their hypo-immunogenicity and paracrine effects after differentiation remains an open question requiring further investigations to be unraveled. Also, would it be better to transplant undifferentiated WJ-MSCs or differentiated IPCs as a cell therapy for DM is an open question waiting further elegant well-designed studies to be answered. Additionally, the long-term effects resulting in either cases after transplantation also require further investigations to unravel for how long exactly would the transplanted cells remain efficient and therapeutically effective? For how long will the genetic stability, low immunogenicity, and safety of those transplanted cells be maintained?

Another recent intervention for treating DM is “Stem Cell Educator” therapy. It is one of the relatively recent modalities/interventions being investigated in phase I/II clinical trials for its safety and efficacy to treat DM, or at least improve insulin resistance. For such intervention, a patient’s blood is circulated through a closed-loop system that separates MNCs from the patient’s whole blood. It briefly co-cultures those MNCs with adherent UCB-derived multipotent stem cells (CB-SCs), and then returns those educated autologous cells back to the patient’s blood circulation (Zhao et al., 2013). Given all the remarkable properties of WJ-MSCs, it is indeed tempting to wonder whether they should be also considered to be utilized for this “Stem Cell Educator” intervention like their counterparts CB-SCs.

Finally, the field of extracellular vesicles, especially those derived from MSCs is expanding rapidly. It was suggested that transplanted MSCs could mediate their beneficial therapeutic effects at least partially via secreting various extracellular vesicles such as exosomes which carry several components such as soluble factors and miRNAs (Leibacher and Henschler, 2016). Undoubtedly, future studies are required to better characterize these vesicles and to help further elucidate the therapeutic potential of these WJ-MSCs-derived extracellular cargos.

## Conclusion

Owing to their unique properties, we believe WJ-MSCs will be frontiers in stem cell therapy. However, several challenges are hindering the transfer of these WJ-MSCs from bench to bed-side. Further elegant large scale well-designed pre-clinical and clinical studies are urgently required to overcome those challenges and sharpen this potential weapon for our battle against DM.

Now understanding their great potential for regenerative medicine, we would recommend WJ-MSCs banking like their counterparts isolated from UCB (for both normal and cesarean labor, at least in hospitals). Actually, given the large number of child-births worldwide, UC provide an unlimited/untapped source of MSCs. Taking in consideration the ever-growing list of advantages and therapeutic effects of WJ-MSCs, not only for DM, but also for various disease conditions, banking of WJ-MSCs and/or UC tissues would indeed turn those UCs from just medical wastes into valuable priceless therapeutic tools. Besides, the medical benefit, this will undoubtedly have a huge economic benefit. We believe, the near future will unravel more interesting findings for WJ-MSCs, and strongly recommend their banking especially for those having family history of DM.

## Author Contributions

The authors declare that they collected the data and wrote the entire article themselves. MK and DK did not receive any form of sponsorship or honorarium for the material, and equally contributed in writing the current review article.

## Conflict of Interest

The authors declare that the research was conducted in the absence of any commercial or financial relationships that could be construed as a potential conflict of interest.
